# Umbilical artery thrombosis diagnosed at different gestational ages and fetal outcomes: a case series

**DOI:** 10.1186/s12884-021-04264-9

**Published:** 2021-11-22

**Authors:** Jing Wei, Qiaoyun Li, Hongbo Zhai

**Affiliations:** 1 Department of obstetrics and gynecology, Affiliated Hangzhou First People’s Hospital, Zhejiang University School of Medicine, Zhejiang, 310006 China; 2 Department of Pathology, Affiliated Hangzhou First People’s Hospital, Zhejiang University School of Medicine, Zhejiang, 310006 China

**Keywords:** Pregnancy, Umbilical artery thrombosis, Umbilical cord thrombosis, Umbilical cord abnormality

## Abstract

**Background:**

Umbilical artery thrombosis is a rare complication of pregnancy strongly associated with poor fetal and perinatal outcomes, such as intrauterine asphyxia, fetal growth restriction, and stillbirth. Its pathogenesis remains unclear, and there is the added challenge of selecting an appropriate delivery time to achieve excellent neonatal outcomes.

**Methods:**

Our Hospital is a critical maternal rescue center with approximately 7000 births annually. We present a series of 8 cases of umbilical artery thrombosis diagnosed at the hospital between Apr 1, 2018, and Jan 31, 2020. We identified the cases through a keyword search of the maternity and pathology information management systems.

**Results:**

Three patients were diagnosed with a transabdominal ultrasound scan and hypoxia on fetal heart monitoring. All three patients had emergency cesarean section delivery. Four patients were observed closely for 5 to 13 weeks from initial detection by an ultrasound scan to delivery. Only one patient was diagnosed after vaginal delivery by Hematoxylin-eosin staining of umbilical cord sections. Seven patients had deliveries by cesarean section, and one patient had a vaginal delivery. All infants were born alive.

**Conclusions:**

Umbilical artery thrombosis is a challenging and rare condition that can occur at different gestational ages, especially when diagnosed in the third trimester and accompanied by fetal growth restriction. Consequently, these patients require close monitoring of umbilical blood flow and fetal growth and intervention at the appropriate time to achieve an optimal outcome.

## Introduction

Umbilical artery thrombosis is an infrequent pregnancy complication, with an estimated incidence of 0.0025 to 0.045% of all gestations [1]. It is clinically easily detectable by Doppler ultrasonographic flow studies and confirmable by postpartum pathological examination of the umbilical cord. Dicke et al. noted that abnormal Doppler ultrasound waveforms occur with umbilical artery thrombosis before obvious fetal impairment [2]. Although it rarely happens, it carries a poor fetal and perinatal prognosis [3]. Shilling et al. reported a series of 7 cases with umbilical artery thrombosis in which two infants were stillborn, and three were small for dates [3]. All the live-born infants had difficult neonatal periods. Following a diagnosis, the challenge remains when best to deliver the baby. Li et al. reported one case in which fetal movements disappeared, and fetal death occurred in utero 2 days after a diagnosis of umbilical artery thrombosis [4]. The objective of this study was to explore the clinical management of umbilical artery thrombosis to enhance fetal outcomes and report our experience.

## Materials and methods

Our hospital is a critical maternal rescue center with approximately 7000 births per annum, and accept all kinds of high-risk pregnant women referred from other hospitals. In the past 10 years, tens of thousands of high-risk pregnant women have been successfully treated, covering various types of obstetrics and medical and surgical critical illnesses. The hospital’s research ethics committee approved the study.

### Data extraction

We identified the 8 cases reported in this paper through a search of the electronic maternity discharge summary records using the search term “umbilical artery thrombosis.”

Between April 2018 and January 2020, we identified 8 cases of umbilical artery thrombosis out of approximately 8400 deliveries.

### Inclusion and exclusion criteria

All patients included in this study had thrombosis of only one umbilical artery by examining Hematoxylin-eosin (H&E) slides of umbilical cord sections. Data extracted from the electronic records for each case included maternal and infant clinical data, Doppler ultrasound results, placental and umbilical cord histological and gross findings, and the infant’s birth situation. We excluded any patient with incomplete data, or concurrent arteriovenous thrombosis.

Two experienced placental pathologists reviewed the H&E slides of all 8 cases. The length, spiral number, and attachment of each umbilical cord were recorded. The umbilical cord abnormalities assessed were marginal or velamentous insertion, stricture, excessively long cord (100 cm or beyond), excessively short cord (30 cm or less), and torsion of the cord (11 circles or more). Fetal growth restriction (FGR) was defined as an estimated fetal weight below the 10th percentile, and severe FGR was defined as an estimated fetal weight below the 3rd percentile.

## Results

In this study, 8 cases were identified and their records retrieved. Ultrasound findings and umbilical artery Doppler studies were available for all eight patients. Clinical data and placental details are shown in Table 1.

**Table 1:** Clinical data and placental details

NO	Diagnosis gestational weeks	Delivery gestational weeks	Obstetric complications	Cord findings	Placental findings	Fetus/neonate
1	32+	32+	Chronic nephritis, polyhydramnios	Thrombosis, cyst, Stained yellow	Chorioamnionitis I	Fetal distress /Apgar 5–7-8 (1790 g), respiratory failure, 27 days in NICU
2	35+	35+	Fever, hypothyrodism, *Listeria monocytogenes* infection	thrombosis	Chorioamnionitis II	Intrauterineasphyxia, Apagr3–7-8 (2220 g) infection; meconium-stained amniotic fluid, septicemia, 2-week stay in NICU
3	24+	37+	Normal	thrombosis	Normal	Apgar 10–10 (2500 g)
4	30+	35+	Preeclampsia, polyhydramnios	Hypercoiling, true knot, velamentous cord insertion	Normal	FGR, Apgar 9–9 (1630 g)
5	37+	37+	polyhydramnios	thrombosis	Normal	Apgar 9–10 (2880 g)
6	postpartum	36+	ICP, viral hepatitis B	Thrombosis	Small (12 cm*12 cm*3 cm)	FGR, Apgar 10–10 (1910 g)
7	24+	34+	Normal	Hypercoiling, thrombosis	Normal	FGR/Apgar10–10 (1300 g),27-day stay in NICU, hyperbilirubinemia
8	24+	35+	ICP	Hypercoiling, thrombosis	Normal	FGR/Apgar10–10 (2000 g), one-week stay in NICU, hyperbilirubinemia

### Maternal characteristics

In these cases, two mothers had no significant medical history. Others had pregnancy complications, including chronic nephritis with proteinuria, hypothyroidism, severe preeclampsia, intrahepatic cholestasis of pregnancy (ICP), high fever, and Listeria infection.

Three of the eight patients underwent amniocentesis for karyotyping and single nucleotide polymorphism microarray **(**SNP) examination because of advanced maternal age or suspected fetal chromosomal abnormality, but amniocentesis revealed no abnormality. The other five patients underwent routine prenatal screening testing, which all suggested low-risk pregnancies.

### Newborn characteristics

All the infants were live born. Two neonates had asphyxia, with one having Apgar scores of 5 and 7 at 1 and 5 min, and the other with Apgar scores of 3 and 7 at 1 and 5 min. Four neonates had birth weights below the 3rd percentile.

**Fig. 1:**
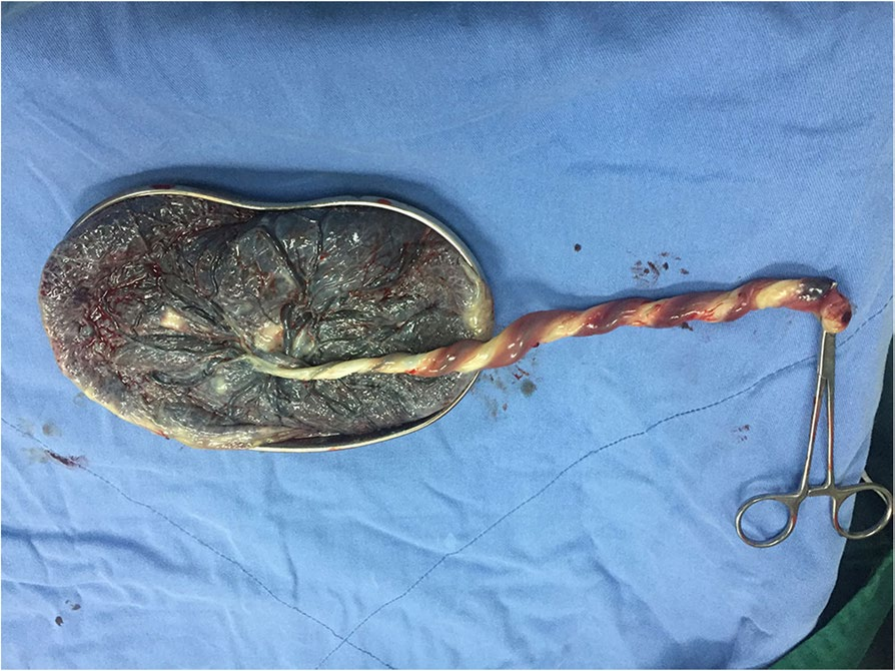
One umbilical artery with a brownish color (case 1)

Four cases had cord abnormalities comprised of coiling abnormalities, including hypercoiling, true knot, and velamentous cord insertion. In addition, in case 1, the umbilical cord had a stricture located 2 cm from the placental insertion site and one umbilical artery with a brownish color suggesting thrombosis (Fig. 1).

**Fig. 2:**
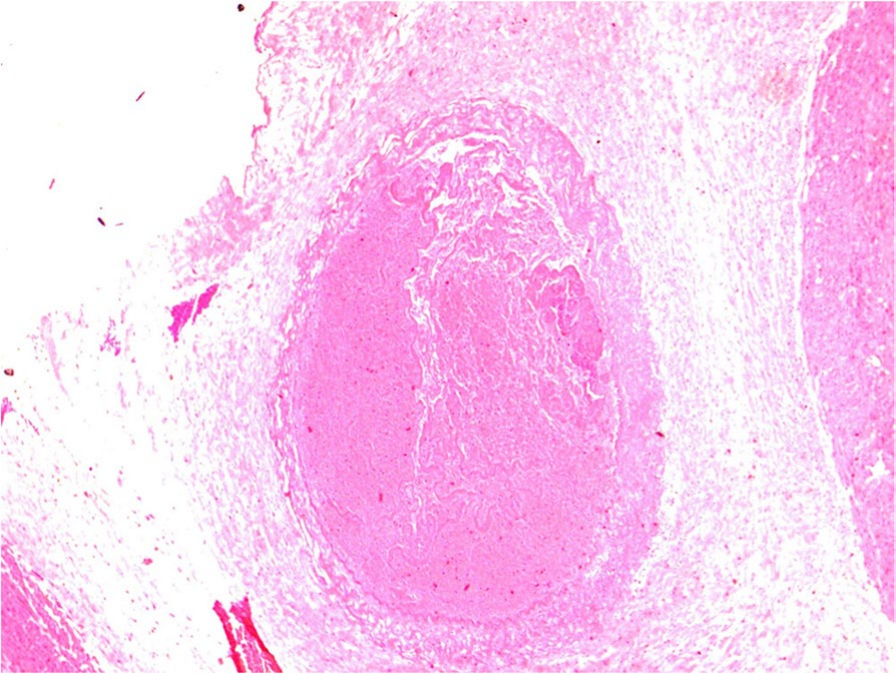
Fibrinoid necrosis in the arterial wall. The arterial lumen is filled with thrombi. Hematoxylin and Eosin, × 20 magnification (case 2)

**Fig. 3:**
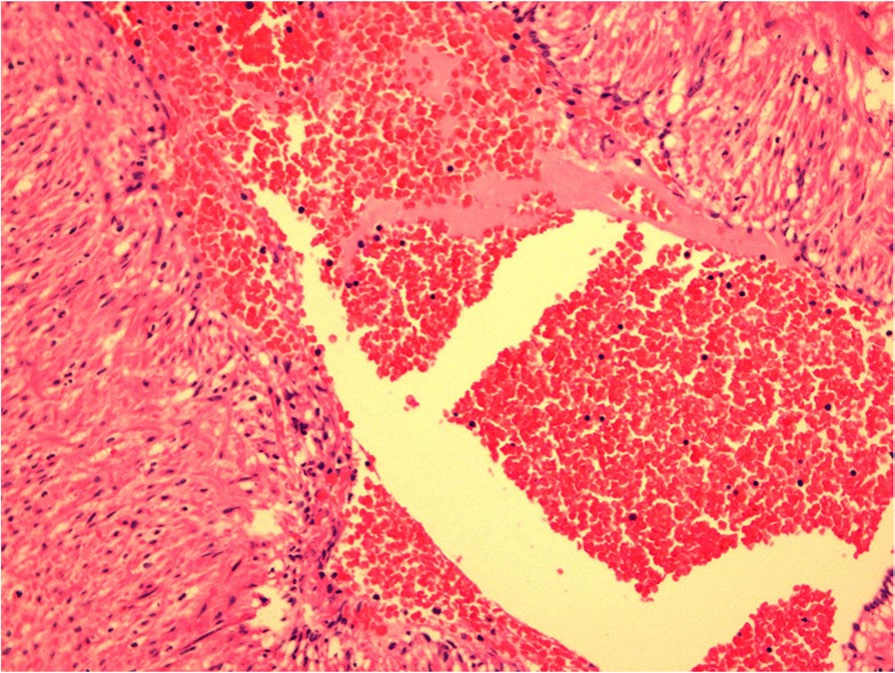
The arterial wall survived. The thrombus is composed of mainly Platelets. Hematoxylin and Eosin, × 40 magnification (case 1)

In all cases, microscopic examination revealed that only one umbilical artery harbored complete occlusive thrombi, and fibrinoid necrosis was observed at the arterial wall (Fig. 2). The other 7 cases exhibited focal thrombi, and the arterial wall survived (Fig. 3). Evidence of chorioamnionitis was present in 2 patients.

## Discussion

Thrombosis of the umbilical cord is extremely rare, with an estimated incidence of 0.0025 to 0.045% of all gestations [1]. This study identified 8 cases of umbilical artery thrombosis out of approximately 8400 deliveries, which corresponds to an incidence of 0.8%. This incidence is higher than those reported in the literature, probably due to our hospital being a tertiary-level facility for critically ill pregnant women. Three of the eight patients were transferred from other hospitals. In 1988, Heifetz [5] identified 52 cases of umbilical cord thrombosis and reported that the prevalence of cord thrombosis is approximately 1 in 1300 deliveries, 1 in 1000 perinatal autopsies, and 1 in 250 high-risk gestations. Avagliano [6] diagnosed 32 cases (10.1%) of umbilical vessel thrombosis in 317 autopsies of spontaneous intrauterine fetal death.

Although different anatomical conditions of the umbilical cord and maternal or fetal pathologies are considered risk factors, the etiology of umbilical cord thrombosis remains unclear in many cases. However, according to Virchow’s hypothesis, thrombosis is related to 3 risk factors: blood stasis, endothelial injury, and hypercoagulability [7]. Most cases of umbilical cord thrombosis in the literature are associated with umbilical cord abnormalities [8–11]. Umbilical cord abnormalities (long cords, short cords, knots, hypercoiling, hypocoiling, strictures, cystic and vascular malformations, and abnormal cord insertion such as velamentous and furcate insertions) can easily cause umbilical cord compression, leading to a reduction of blood flow and consequent thrombosis. Chew [8] reported a case of intrauterine fetal demise of a fetus with multiple umbilical cord strictures and vascular thrombosis. Oliveira [9] reported a case of severe intrauterine growth restriction associated with a long, thin, and overly twisted umbilical cord. We found four patients with umbilical cord abnormalities in our series, including hypercoiling, true knots, velamentous insertions, strictures, and cystic and vascular malformations.

Materno-fetal infections and some obstetric complications may also cause endothelial damage to the umbilical cord. In this study, one mother had a high fever and subsequently tested positive for *Listeria monocytogenes*. Moreover, two patients with Chorionamnionitis I-II were identified. Inflammation can lead to vascular endothelial injury, with possible pathogenesis of umbilical artery thrombosis.

Hypercoagulability may be associated with inherited or acquired maternal or fetal thrombophilia. Alhousseini [12] reported a case of umbilical artery thrombosis with associated acute and severe fetal growth restriction. The baby was found to have transient severe protein S deficiency at birth, which was later resolved at 2 months of age. The author [12] speculated that this deficiency was present before delivery and may have played a role in the thrombosis of one of the umbilical arteries. However, in our series, maternal and neonatal coagulation parameters and D-dimer levels were normal. Roopali et al. reported a case of umbilical artery thrombosis after intrauterine transfusion due to severe fetal anemia secondary to Rh alloimmunization [13].

Thrombosis of the umbilical artery is strongly associated with fetal morbidity and mortality. In the literature [1, 3, 4, 8–12, 14], 27 cases of umbilical cord thrombosis with fetal outcomes were reported. Of these cases, 6 (22%) were stillborn, 11 (40%) had FGR, and 2 (7%) suffered fetal distress. In our study, all neonates were born alive, with 2 cases of fetal distress and 4 cases of FGR. The neonate in case 1 was admitted to the neonatal intensive care unit for 27 days because of respiratory failure, and the neonate in case 2 had a 2-week stay in the neonatal intensive care unit because of septicemia.

Three mothers underwent emergency cesarean section at 32–37 weeks gestational age when the ultrasound revealed an absence of blood flow in one of the two umbilical arteries. There was a disappearance of baseline variability in fetal heart rate and decreased fetal movement.

Four patients were observed for 5 to 13 weeks from the first detection by ultrasound to delivery with close follow-up. Case 3 was first identified at 24 weeks gestation, with one of the two umbilical artery absent by color Doppler examination. We followed up the patient with daily fetal heart monitoring at home and weekly ultrasound scans in our hospital. At 37 weeks gestation, the mother presented with premature rupture of membranes. Induction of labor was followed by a normal vaginal delivery of a healthy infant weighing 2500 g. Case 4 was first identified by color Doppler examination at 30 weeks gestation. She was closely followed up and underwent an emergency cesarean section at 35 weeks gestation because of acute fetal distress indicated by a non-stress test. The neonatal Apgar score was 9 at 1 min, and birth weight was below the 3rd percentile for gestational age. Cases 7 and 8 were first detected with an absence of blood flow in one of the two umbilical arteries and estimated fetal weights below the 10th percentile at 24 weeks of gestation. We informed both patients of the pregnancy risks and observed fetal development and umbilical cord blood flow with close follow-up. Cases 7 and 8 were given low molecular weight heparin 4100 IU per day from 24 weeks gestational age until the day of cesarean section. This is a trial treatment based on the preventive effect of low molecular weight heparin on thrombosis and its safety during pregnancy. Case 7 underwent an emergency cesarean section at 34 weeks gestation when fetal heart monitoring showed prolonged deceleration. Case 8 underwent an elective cesarean section at 35 weeks gestation when her total bile acid was more than 40umol/L. The two neonatal Apgar scores were 10 at 1 min, and birth weights were below the 3rd percentile.

In only one case (case 6), ultrasonography indicated fetal growth restriction below the 3rd percentile but without an abnormal umbilical cord blood flow. At 36 weeks gestation, the mother went into labor and delivered an infant weighing 1910 g. H&E slides confirmed the diagnosis of umbilical artery thrombosis.

As seen from the above cases, we performed emergency cesarean sections when abnormal fetal heart monitoring occurred during the third trimester. This is consistent with previous reports that support the more effective approach of emergency cesarean section during the third trimester [15]. With good fetal heart monitoring and fetal growth, vaginal delivery may be an option, but an elective cesarean section may be more appropriate when cessation of fetal growth occurs [9].

## Conclusions

Thrombosis of the umbilical artery is an infrequent but clinically significant pregnancy complication, with a poor perinatal prognosis. The absence of one umbilical artery is identifiable by dynamic Doppler flow studies and comparison of prenatal ultrasound scans. This paper discusses for the first time the management of the fetus with a differential diagnosis of umbilical artery thrombosis, choice of delivery mode, and prognosis of the fetus. When umbilical artery thrombosis is found at term or near term, with abnormal fetal heart monitoring, an emergency cesarean section can often obtain a better outcome. When umbilical artery thrombosis is found in the second trimester and accompanied by fetal growth restriction, the management of the pregnancy becomes challenging. The limitations of this study are the small number of cases and the lack of long-term neurological and cognitive follow-up of the neonates. Fetal growth and umbilical cord blood flow should be monitored closely, and delivery performed at the most appropriate time to enhance neonatal outcomes. The prophylactic use of low molecular weight heparin in these pregnancies needs further evaluation.

## Data Availability

The datasets used and/or analyzed during this study are available from the corresponding author on request.

## Abbreviations

FGR: Fetal growth restriction
H&E: Hematoxylin-eosin
ICP: Intrahepatic cholestasis of pregnancy
SNP: Single nucleotide polymorphism microarray
